# V1 and V2 pericordial leads misplacement and its negative impact on ECG interpretation and clinical care

**DOI:** 10.1111/anec.12844

**Published:** 2021-04-04

**Authors:** Anis Abobaker, Rehman Mehdi Rana

**Affiliations:** ^1^ Spire Fylde Coast Hospital Blackpool UK; ^2^ York Teaching Hospital NHS Foundation Trust Scarborough UK

Dear Editor,

The correct placement of V1 and V2 pericordial leads of the electrocardiogram (ECG) is on the fourth intercostal space to the right and the left margins of the sternum (Walsh, 2020). Misplacement of V1 and V2 leads where leads are placed higher than the fourth intercostal space is a common technical error in clinical practice (Walsh, 2018). Unfortunately, this has a negative impact on ECG interpretation as it could generate few false‐positive findings such as old septal myocardial infarction (MI), acute anterior ST‐segment elevation myocardial infarction (STEMI) pattern, and incomplete right bundle branch block (RBBB) (Walsh, 2018). The misinterpretation of the false ECG findings could lead to unnecessary medical management and intervention (Ilg and Lehmann, 2012). Interestingly, the morphology of P wave in V1 and V2 is a key factor which could help the physicians to differentiate between leads misplacement and true ECG findings. When V1 and V2 leads are placed correctly, in majority of healthy individuals P wave in V1 has a biphasic morphology (Walsh, 2020), whereas in V2 the shape of P wave is expected to be upright (Ilg and Lehmann, 2012). When V2 lead is placed higher than the fourth intercostal space, the positive amplitude of the P wave in V2 can be lost, and abnormal P wave shapes can be observed, such as flat, biphasic, and negative P waves based on the degree of the lead misplacement (Ilg and Lehmann, 2012). Furthermore, a predominantly negative P wave in V1 should raise the suspicion of V1 and V2 leads misplacement (García‐Niebla et al., 2012).

Herein, we present three ECG examples done in the preassessment clinic at Spire Fylde Coast Hospital for patients undergoing elective surgical procedures where misplacement of V1 and V2 leads generated false‐positive findings (Figure 1) resulted in delay of their surgical procedures pending further cardiological assessment. When the ECGs were repeated after careful and correct placement of V1 and V2 leads, all the ECG findings were fully resolved. In all the three examples, the P wave shape in V1 was predominantly negative, whereas in V2 the positive amplitude of P wave was lost. When the leads are placed correctly, biphasic, and positive, P waves in V1 and upright P waves in V2 can be clearly seen in addition to full resolution of the false‐negative ECG findings.

**FIGURE 1:**
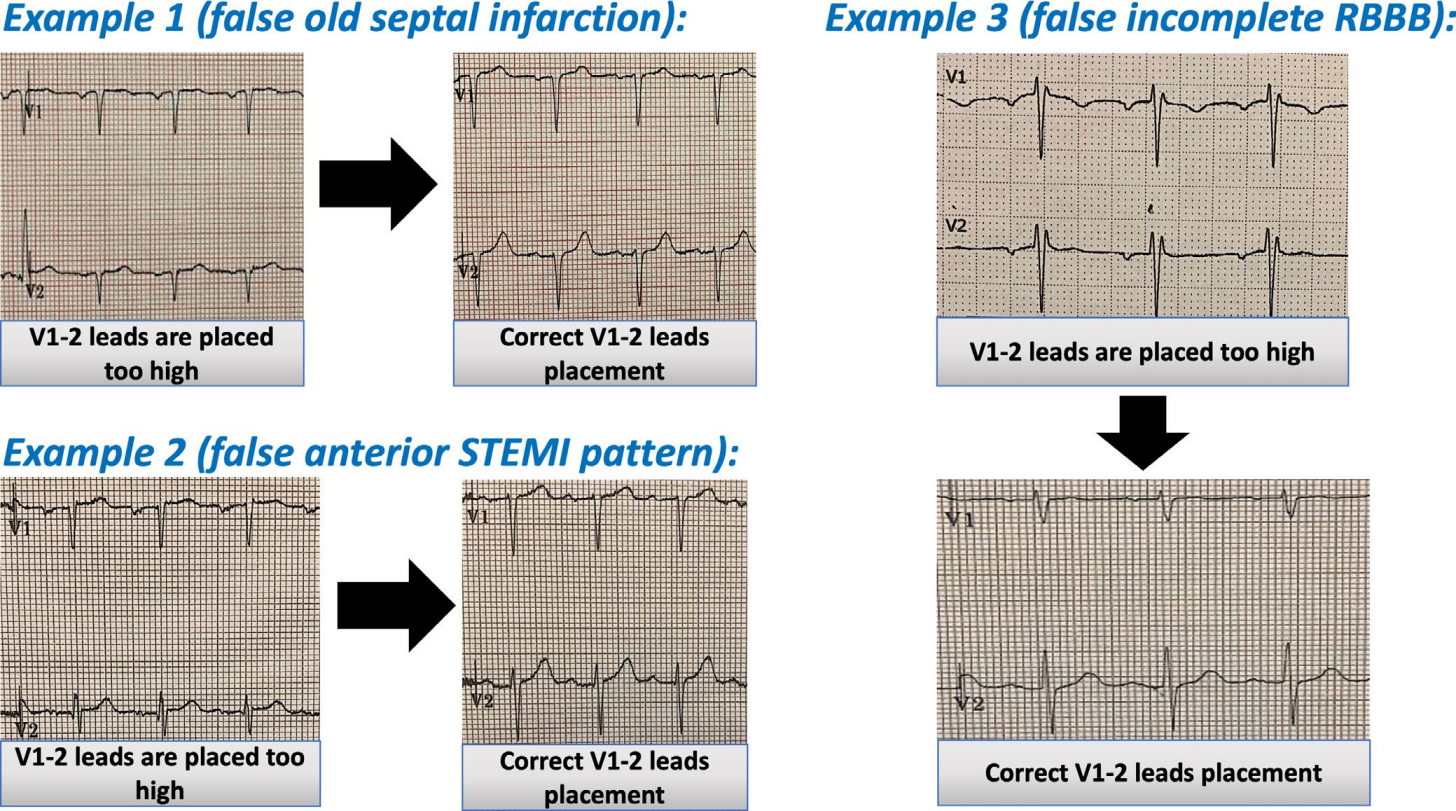
Three real examples of false‐positive ECG findings generated by V1 and V2 leads misplacement. Example 1, incorrect V1‐2 leads placement generated septal Q waves which was misinterpreted as old septal infarction. After correct leads placement, small R waves in V1 and V2 can be seen and septal Q waves disappeared. Example 2, shows Subtle ST elevation in V1 and V2, which was misinterpreted as a possible anterior MI, the ST‐segment elevation in V1 and V2 resolved after correct V1‐2 leads placement. Example 3, shows RsR pattern in V1 and V2 suggestive of incomplete RBBB. Following correct V1‐2 placement, normal RS pattern in V1 and V2 can be seen

In conclusion, the main learning point of presenting these ECG examples is to raise the awareness of the importance of careful and correct pericordial leads placement when performing the ECG as pericordial leads misplacement has negative consequences which could affect accurate ECG interpretation and the quality of care delivered to the patients. Furthermore, the morphology of P wave in V1 and V2 is a useful tool to detect pericordial leads misplacement so physicians can avoid ordering further investigations or interventions unnecessarily.

## CONFLICT OF INTEREST

The authors declare that they have no conflict of interest.

## References

[anec12844-bib-0001] García‐Niebla, J. , Rodríguez‐Morales, M. , Valle‐Racero, J. , & de Luna, A. (2012). Negative P wave in V1 Is the Key to Identifying High Placement of V1–V2 Electrodes in Nonpathological Subjects. The American Journal of Medicine, 125(9), e9–e10. 10.1016/j.amjmed.2011.12.024 22938933

[anec12844-bib-0002] Ilg, K. , & Lehmann, M. (2012). Importance of Recognizing Pseudo‐septal Infarction due to Electrocardiographic Lead Misplacement. The American Journal of Medicine, 125(1), 23–27. 10.1016/j.amjmed.2011.04.023 21851916

[anec12844-bib-0003] Walsh, B. (2018). Misplacing V1 and V2 can have clinical consequences. The American Journal of Emergency Medicine, 36(5), 865–870. 10.1016/j.ajem.2018.02.006 29472037

[anec12844-bib-0004] Walsh, B. (2020). Misplacement of V1 and V2 • LITFL • ECG Library Basics [Internet]. Life in the Fast Lane • LITFL • Medical Blog. 2020 [cited 24 January 2021]. Available from: https://litfl.com/misplacement‐of‐v1‐and‐v2/?fbclid=IwAR09XYsqyJDaGzVDitmPNyfsOcls5ixNAyTnnTX74TwsOErY1KFZzgi‐SR4

